# Delayed presentation of splenic artery pseudoaneurysm: A critical outcome of blunt abdominal trauma; A case report

**DOI:** 10.1016/j.ijscr.2024.109799

**Published:** 2024-05-23

**Authors:** Ahmed Almumtin, Mohamed Ouhlous, Madawi Alsharhan, Afnan Ahmed, Inaam Ahmed Ibrahim, Isam Osman

**Affiliations:** a King Faisal Specialist Hospital and Reseach Center, Riyadh, Saudi Arabia; b King Saud medical city, Riyadh, Saudi Arabia; c Alfaisal university, Riyadh, Saudi Arabia

**Keywords:** Splenic artery, Pseudoaneurysm, Blunt trauma, Endovascular, Embolization, Case report

## Abstract

**Introduction and importance:**

Blunt abdominal trauma is one of the most common reasons for emergency department visits, and spleen and splenic vasculature is involved variably in those cases. Splenic artery pseudoaneurysm formation is one complication with potentially devastating consequences. Early detection and management are of paramount importance given its potential fatality. Management includes open repair with or without splenectomy, and endovascular approach. The minimally invasive endovascular treatment offers earlier recovery, preserved splenic function, and positive outcomes. We report a case of delayed presentation of a large splenic artery pseudoaneurysm after blunt abdominal trauma, managed using endovascular intervention.

**Case presentation:**

A 45-year-old male presented 10 days after being involved in a pedestrian accident with blunt abdominal trauma resulting in a large splenic artery pseudoaneurysm. After multidisciplinary discussion, the decision was to take him for endovascular treatment. The patient recovered very well and was discharged two days later and followed up in an outpatient setting. Over a year, he became symptom free, and demonstrated radiological finding of shrinking pseudoaneurysm.

**Clinical discussion:**

Pseudoaneurysms of visceral arteries are repaired regardless of their size per society of vascular surgery guidelines. Larger ones are at higher risk of rupture and are associated with high mortality. When discovered, treatment plans should be readily discussed, and undertaken. In our case, the patient had a 6.5 cm splenic artery pseudoaneurysm, and a multidisciplinary meeting was conducted and concluded that endovascular treatment would be the best modality to start with, with surgical option as a backup in a hybrid room setting.

**Conclusion:**

Blunt abdominal trauma can present with overt symptoms of internal organ injury; however, some might be missed and need high index of suspicion and therefore further testing and imaging. Splenic artery pseudoaneurysms can expand and rupture in delayed presentation, early detection and management is of paramount importance. Endovascular treatment represents an excellent modality, with minimal invasive nature, faster recovery, and early return to daily activity with preserved splenic function.

## Highlights

• Blunt abdominal trauma poses a risk of injury to the spleen and splenic artery.
• Pseudoaneurysms carry a significantly elevated risk of mortality when they rupture.
• Safe management requires a comprehensive assessment, and high index of suspicion for delayed trauma-related events.
• Endovascular surgery is minimally invasive, proves effective in proficient hands and linked to favorable outcomes.
• Essential for optimal care are hybrid rooms equipped with the right instruments and expertise.

## 1 Introduction

Blunt abdominal trauma, frequently associated with motor vehicle crashes, is a common cause of emergency hospital visits [1]. Higher-impact traumas result in varying degrees of intra-abdominal injuries with the spleen being the most frequently injured organ, comprising over 60 % of cases, followed by liver and hollow viscus injuries [2]. Classification systems such as the American Association for the Surgery of Trauma (AAST) and the World Society of Emergency Surgery (WSES) focus on parenchymal and hilar vascular splenic injuries but tend to look past isolated splenic artery injuries [3].

Splenic trauma poses a significant risk of rapid hemorrhage, prompting immediate investigations [4]. Intervention is recommended for the management of all visceral artery pseudoaneurysms, according to the 2020 guidelines from the Society of Vascular Surgery, due to their susceptibility to rupture including splenic [5]. Management strategies, which include non-operative and operative approaches, are based on the patient's hemodynamic stability [4]. Computed Tomography Angiography (CTA) is considered the gold standard for visualization of splenic injuries including splenic artery pseudoaneurysms, facilitating early diagnosis, and allowing proper planning of treatment [6]. Moreover, non-operative interventions such as an endovascular approach are preferred over operative due to their minimally invasive nature and association with favorable outcomes when done skillfully, making it a better choice overall [7].

Herein we report a case of a patient who was treated endovascularly after presenting with symptoms of pseudoaneurysm 10 days after being in a car accident as a pedestrian.

## 2 Case presentation

A 45-year-old male with an insignificant medical history presented with a complaint of worsening left upper abdominal pain over ten-days prior to presentation. His symptoms started after he was struck by a car in an accident. He reported no previous episodes of similar pain and denied having any nausea or vomiting. Upon examination, the patient exhibited normal vital signs, the abdomen was soft to palpation with tenderness and non-pulsatile swelling in his epigastrium. No other injuries were appreciated apart from abrasions in his upper and lower limbs.

**Fig. 1:**
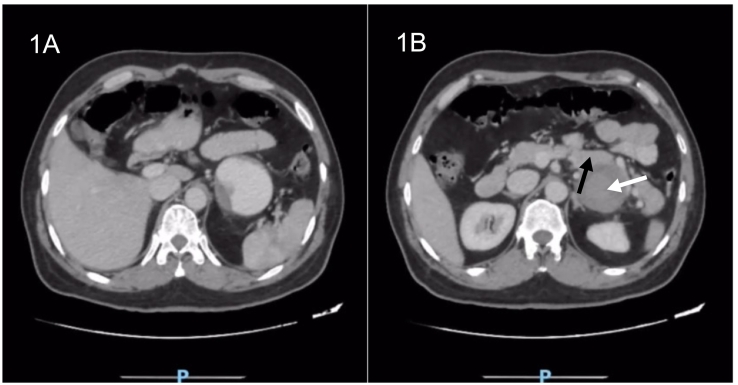
Pre-embolization. 1A: Contrast filled splenic artery pseudoaneurysm. 1B: Splenic artery pseudoaneurysm (white arrow) arising from the middle of the splenic artery (black arrow).

Initial blood samples revealed a hemoglobin level of 15 g/dL, with normal coagulation and renal panels. Initial Focused Assessment Sonography in Trauma (FAST) was negative for intraperitoneal free fluids. A subsequent CTA revealed a partially thrombosed 6.5 cm pseudoaneurysm situated between the mid and distal portions of the splenic artery. Additionally, the inlet of the aneurysm was not clearly discernible in the CTA images (Fig. 1).

In response to these findings, a multidisciplinary meeting involving vascular, trauma surgery and interventional radiology was convened. The consensus reached was to proceed with the patient to the hybrid room for angiography and embolization considering the patient was hemodynamically stable and no other concomitant injuries necessitating a laparotomy, with the possibility of an immediate conversion to celiotomy should it rupture. In preparation, blood products were made available, and the patient was informed about expectations and potential complications associated with the procedure.

## 3 Surgery

**Fig. 2:**
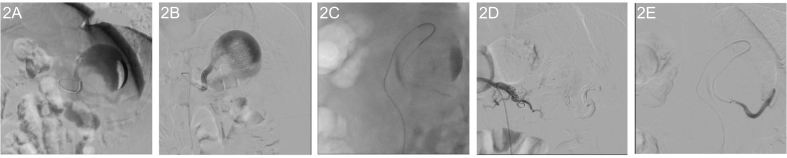
Fluoroscopy images. 2A: Fluoroscopic image of the splenic artery pseudoaneurysm. 2B: Fluoroscopic image capturing the injection of Glubran-lipiodol solution into the splenic artery pseudoaneurysm. 2C: Successful cannulation of distal splenic artery. 2D: Intraoperative fluoroscopic image showing the placement of coils in the proximal splenic artery, resulting in the complete exclusion of the splenic artery pseudoaneurysm. 2E: Angiogram of the distal splenic artery (the inlet).

The patient was taken to the operating room, under local anesthesia and sedation, right femoral artery was punctured, 5 French sheath was inserted. Using a 0.035 hydrophilic Glidwire® (Terumo) and a 4fr hydrophilic coated Berenstein catheter were used to navigate to the proximal splenic artery. Angiogram was done (Fig. 2A), revealed a saccular splenic artery pseudoaneurysm with partial thrombosis. Multiple attempts to land the wire in the distal splenic artery were made but were initially unsuccessful. At this stage we decided to inject the pseudoaneurysm with a solution of Lipidol and cyanoacrylateCa (GEM, Viareggio, Italy), to obliterate the PSA. The embolization mixture was injected into the aneurysm, but unfortunately settled in the bottom and lateral part of the aneurysmal sac (Fig. 2B). Using TrueSelect™ microcatheter (Boston scientific) to navigate into the distal splenic artery within the Bern catheter, with no success. Finally, we exchanged the Berenstein catheter for a Cobra catheter, and again with TrueSelect™ we were able to reach the distal splenic artery (Fig. 2C). 3 ml of the embolization mixture was enough to embolize the distal splenic artery. VortX Pushable coils (Boston scientific) and Interlock eighteen were placed in the inlet (Fig. 2D). The angiogram done at the conclusion of the procedure revealed a completely excluded aneurysm, with no distal splenic artery flow (Fig. 2E). The puncture site was controlled by pressure for twenty minutes. The patient tolerated the procedure well and shifted to recovery in a stable condition.

## 4 Post operative

**Fig. 3:**
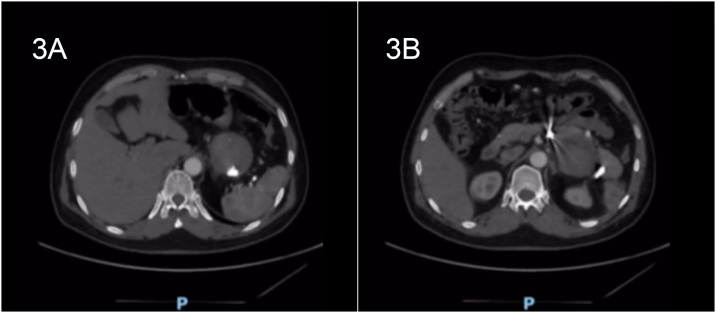
Upon discharge, the pseudoaneurysm size (6.4 x 6.9 cm) remains unchanged, indicating a successfully embolized splenic artery pseudoaneurysm with no contrast filling.

**Fig. 4:**
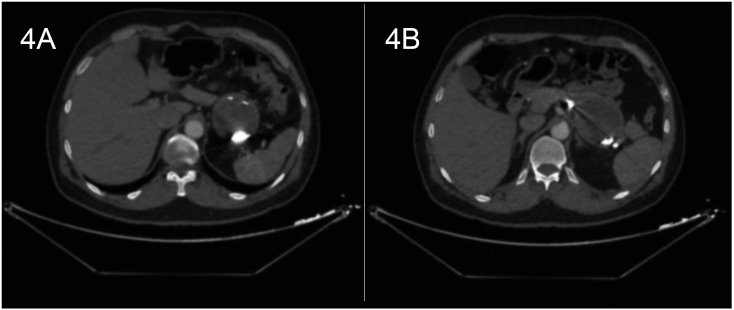
7 months post-embolization; little decrease in the size of the splenic artery pseudoaneurysm (6 x 6 cm).

The patient was kept in the general surgical ward, with serial abdominal exams and vital signs monitoring. He received pneumococcus, *Haemophilus influenzae* type B, and meningococcal vaccines postoperatively upon discharge after consultation with infectious diseases specialist. He had a smooth post-operative course and was eventually discharged after obtaining a CTA (Fig. 3), and outpatient follow-up visits (Fig. 4).

## 5 Follow up

**Fig. 5:**
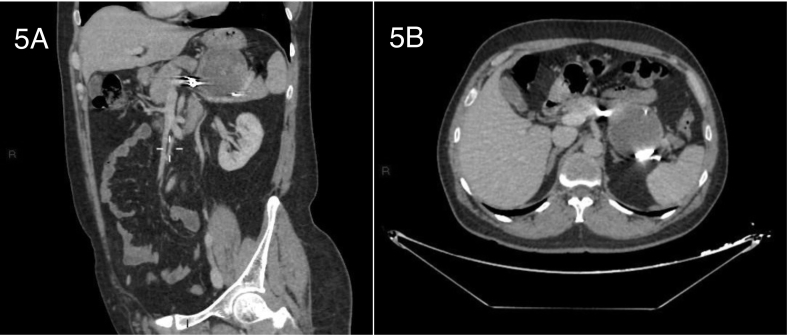
CT images in delayed phase. 5A: CT angiography in coronal cut, showing no flow in the pseudoaneurysm 7 months later. 5B: CT angiography in axial cut, showing no flow 7 months later.

The patient was followed up as an outpatient, had complete symptoms resolution, and followed up with CT angiography. 7 months later CT angiography showed slight shrinkage in the glue mixture filled pseudoaneurysm despite absence of flow into the aneurysmal sac in delayed images (Fig. 5), and perfused splenic parenchyma and given follow up appointments for future ultrasonographic evaluations.

## 6 Discussion

The spleen is commonly affected in cases of blunt abdominal trauma, constituting 25 % of annual admissions in the United States [8]. As it is one of the most vascularized organs, the spleen is prone to substantial blood loss, primarily from arterial sources, leading to intraperitoneal hemorrhage. Trauma to the left lower chest (9th, 10th, and 11th ribs) causes it to be highly susceptible to injury. It is essential to acknowledge potential physiological issues stemming from splenic trauma, as failure to do so can result in devastating outcomes that could be prevented. Moreover, preserving the spleen allows for the retention of its immunological roles [8].

Visceral artery pseudoaneurysm is infrequent as reported by Tessier et al. (2003) in the Journal of Vascular Surgery [9]. They identified thirty-seven cases, ten were related to the splenic artery with 80 % exhibiting symptoms. The study further mentioned that post-traumatic pseudoaneurysms are typically diagnosed during the hospital recovery phase, within the timeframe of 1 day to 4 months (median, 4 days) and symptoms may persist months after injury. The average pseudoaneurysm size was notably smaller compared to what had been reported in literature. Whereas in our case, the size of the pseudoaneurysm measured at 6.5 cm, making it larger than the reported average and the patient presented with abdominal pain only after a period of 10 days of being asymptomatic. Regardless of size or symptoms, it is recommended to repair all identified splenic artery PSAs due to their substantial risk of rupture.

Our approach involved a FAST scan to exclude the presence of free fluid in the peritoneum which would prompt immediate surgical intervention. Following negative results, a CTA scan was ordered to further explore for any other organ injury. CTA is the ideal modality for early detection as it provides timely and detailed imaging which is crucial for influencing the choice between endovascular treatment or surgery of splenic arterial injury [8]. Additionally, it aids in the approach to managing organ damage by assessing density differences between splenic parenchyma and hematoma. The preference of non-operative therapy for blunt splenic injuries raises the possibility of a growing incidence of splenic artery pseudoaneurysms. It remains uncertain whether this potential rise is due to an actual increase in occurrences or enhanced detection facilitated by the frequent use of follow-up CT scans [9].

The mortality rate for patients undergoing endovascular treatment stands at 0.5 %, significantly lower than the 4.9 % observed with open surgery [10]. This can be attributed to post surgical adverse events including pulmonary and abdominal complications (such as atelectasis, pleuritic effusion, pneumonia, intraperitoneal infections, and injury to adjacent organs), fistula formation and most commonly, pseudoaneurysm rupture. Furthermore, splenectomy carries a significant risk of hemorrhage recurrence, abscess formation as well as overwhelming post-splenectomy infection (OPSI) [4,7].

Endovascular techniques offer an alternative approach for treating these aneurysms. The main technique involves filling the aneurysm with embolizing agents and if blood flow remains after the procedure, it is considered unsuccessful. Currently, coils are the primary choice, although other methods such as use of detachable balloons or inert particles have also been implemented. For the treatment of large aneurysms and pseudoaneurysms, a combination of resorbable gelatin (Gelfoam) and coils has proven to be beneficial. Most of the splenic vascularization must be preserved by utilizing collateral vessels from the gastric, omental, and pancreatic regions, therefore, careful selection of the occlusion site must be considered and was achieved in our case [11].

Post-embolization, approximately 20 % of patients experience splenic infarction, leading to the devascularization of 25 % of the spleen [8]. Therefore, continuous abdominal examinations and complete blood count monitoring are crucial, and any signs of hemodynamic instability should promptly be recognized. A few days after embolization, 30 % of patients may experience post embolization syndrome (PES) which is characterized by fever (38 °C), abdominal pain and pancreatitis attacks and typically resolves within 3–5 days [11]. Fortunately, no post-procedural complications occurred in our case and further CTA scans of the patient after discharge revealed intact spleen parenchyma with no infarcts.

## 7 Conclusions

Blunt abdominal trauma can present with overt symptoms of internal organ injury; however, some might be missed and need a high index of suspicion and therefore further testing and imaging. Splenic artery pseudoaneurysms can expand and rupture in delayed presentation, early detection and management is of immense importance. Endovascular treatment is an excellent modality, with minimal invasive nature, faster recovery, and early return to daily activity with preserved splenic function. The patient was stable following initial assessment and was successfully managed by endovascular embolization which was concluded to be the best option as there were no indications for an operative approach.

## Disclaimer

Ethical approval for this study (**H2RI-07-Mar24-01**) was provided by the Research & Innovation Centre at King Saud Medical City – Riyadh – Saudi Arabia. March 12, 2024.

Consent was obtained from the patient to publish this case report including information, pictures, and radiological images. No competing interest to be declared. No conflict of interest to be reported. We received no financial support in publishing this case or in any steps of preparation. No AI function was used.

The case report was reported in line with PROCESS [12] criteria and SCARE checklist [13].

## Consent

Written informed consent was obtained from the patient for publication and any accompanying images. A copy of the written consent is available for review by the Editor-in-Chief of this journal on request.

## Ethical approval

Ethical approval for this study (**H2RI-07-Mar24-01**) was provided by the Research & Innovation Centre at King Saud Medical City – Riyadh – Saudi Arabia. March 12, 2024

## Funding

No funding was received.

## Author contribution

Ahmed Almumtin: Principal author, idea, writing.

Mohammed Ouhlous: Revision and treatment methodology writing.

Madhawi Alsharhan: literature review.

Afnan Ahmed: literature review.

Inaam Ibrahim: literature review.

Isam Osman: Overall supervision.

## Guarantor

Ahmed Almumtin.

## Research registration number

**H2RI-07-Mar24-01**

## Conflict of interest statement

No conflict of interest.
